# Effect of temperature on *Burkholderia pseudomallei* growth, proteomic changes, motility and resistance to stress environments

**DOI:** 10.1038/s41598-018-27356-7

**Published:** 2018-06-15

**Authors:** Suporn Paksanont, Kitisak Sintiprungrat, Thatcha Yimthin, Pornpan Pumirat, Sharon J. Peacock, Narisara Chantratita

**Affiliations:** 1 Department of Microbiology and Immunology, Faculty of Tropical Medicine, Mahidol University, Bangkok, Thailand; 2 London School of Hygiene and Tropical Medicine, London, UK; 3 Mahidol-Oxford Tropical Medicine Research Unit, Faculty of Tropical Medicine, Mahidol University, Bangkok, Thailand

## Abstract

*Burkholderia pseudomallei* is a flagellated, gram-negative environmental bacterium that causes melioidosis, a severe infectious disease of humans and animals in tropical areas. We hypothesised that *B*. *pseudomallei* may undergo phenotypic adaptation in response to an increase in growth temperature. We analysed the growth curves of *B*. *pseudomallei* strain 153 cultured in Luria–Bertani broth at five different temperatures (25 °C–42 °C) and compared the proteomes of bacteria cultured at 37 °C and 42 °C. *B*. *pseudomallei* exhibited the highest growth rate at 37 °C with modest reductions at 30 °C, 40 °C and 42 °C but a more marked delay at 25 °C. Proteome analysis revealed 34 differentially expressed protein spots between bacterial cultures at 42 °C versus 37 °C. These were identified as chaperones (7 spots), metabolic enzymes (12 spots), antioxidants (10 spots), motility proteins (2 spots), structural proteins (2 spots) and hypothetical proteins (1 spot). Of the 22 down-regulated proteins at 42 °C, redundancy in motility and antioxidant proteins was observed. qRT-PCR confirmed decreased expression of *fliC* and *katE*. Experiments on three *B*. *pseudomallei* strains demonstrated that these had the highest motility, greatest resistance to H_2_O_2_ and greatest tolerance to salt stress at 37 °C. Our data suggest that temperature affects *B*. *pseudomallei* motility and resistance to stress.

## Introduction

*Burkholderia pseudomallei* is the causative agent of melioidosis, a severe and often fatal disease of humans and animals that is endemic in tropical regions of Asia and northern Australia^1^. The bacterium can be isolated from soil and water in melioidosis-endemic regions^2–4^. Infection occurs through skin inoculation, inhalation and ingestion and can be difficult to eradicate^1^, and *B*. *pseudomallei* can remain dormant in humans for a prolonged period^1^. In northeast Thailand, melioidosis accounts for approximately 20% of all community-acquired septicaemia and is associated with a 40% mortality rate. The disease has a wide spectrum of clinical syndromes but the major manifestations are sepsis, bacteraemia, pneumonia and abscesses in multiple tissues and organs^1^.

*B*. *pseudomallei* has a large genome compared with those of many other species, with two chromosomes of 4.07 Mbp and 3.17 Mbp^5^. Multiple genes support adaptation and survival in different environments^5^. *B*. *pseudomallei* has been reported to survive in distilled water without nutrients for at least 16 years^6^. Within the infected host, *B*. *pseudomallei* becomes intracellular and can persist in a range of cell types including phagocytic cells, surviving the phagolysosome environment where it is exposed to significant oxidative stress^7^. *B*. *pseudomallei* encodes numerous virulence factors including cell-associated and secretory factors such as type 3 and type 6 secretion systems, proteinases, lipase, lipopolysaccharide, capsule, biofilm and flagella^8^. Many of these are regulated by bacterial and environmental factors including quorum-sensing systems^9,10^, VirAG two-component regulatory system^11^, acid^12^, salt stress^13^ and nutritional availability^14^.

The factors that provide a conducive environment for the survival of *B*. *pseudomallei* in adverse conditions are poorly understood. Many bacterial species have altered expression of stress response proteins and repair enzymes as a strategy to reduce temperature-associated damage^15–19^. This inducible resistance to heat may be essential for bacterial survival in the host. Patients with melioidosis have protracted fever, which is induced by pro-inflammatory mediators produced by the host immune response against bacterial infection. However, it is not known whether high temperature *in vivo* aids bacterial dissemination, chronic infection or inhibits bacterial multiplication. This indicates a need to understand growth and phenotypic adaptations of *B*. *pseudomallei* to heat stress, which requires a discriminatory tool at a proteomic level. We previously used a proteomic approach to characterise and compare protein expression profiles of three different colony morphotypes of *B*. *pseudomallei* and described complex changes associated with morphotype switching, which lends support to the idea that this is associated with a fitness advantage under stress environments *in vivo*^12^.

We hypothesised that *B*. *pseudomallei* undergoes phenotypic adaptation in response to high temperatures *in vivo*. This study aimed to compare the growth rate of *B*. *pseudomallei* cultured at five different temperatures and to use proteomic methods to determine differential protein expression, combined with quantitative reverse transcriptase PCR (qRT-PCR) to confirm protein expression. The effect of different temperatures on phenotypic changes in *B*. *pseudomallei* were also examined, including cell motility, flagellar expression, resistance to oxidative and salt stress and biofilm formation. These data provide novel insights into phenotypic changes in *B*. *pseudomallei* that may be associated with pathogenesis *in vivo*.

## Results

### Effect of temperature on the growth rate of *B*. *pseudomallei*

**Figure 1:**
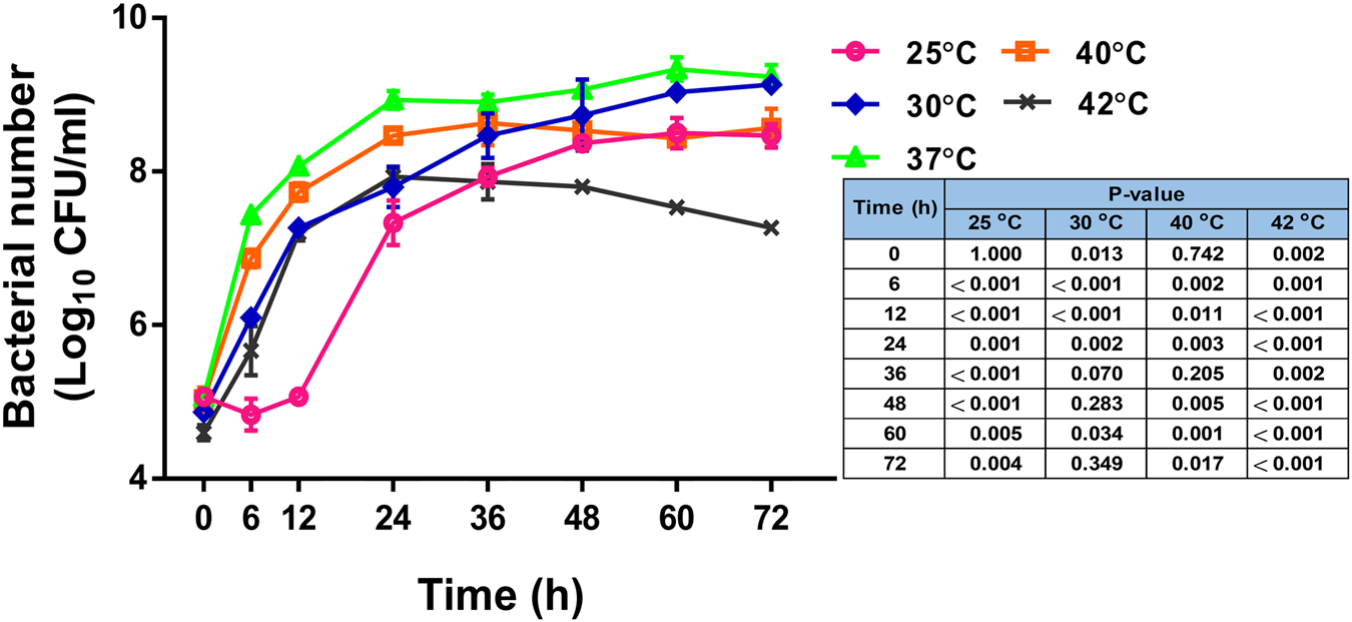
Growth curves of *B*. *pseudomallei* strain 153 in LB broth at 25 °C, 30 °C, 37 °C, 40 °C and 42 °C. P values of bacterial number at different temperatures at different time points relative to 37 °C are shown in the table.

Temperature affects bacterial growth and protein stability^20^. We investigated the effect of different temperatures on the growth rate of a clinical isolate of *B*. *pseudomallei* from a patient with melioidosis, strain 153 in Luria–Bertani (LB) broth at 25 °C, 30 °C, 37 °C, 40 °C and 42 °C. Growth curve analysis revealed that *B*. *pseudomallei* exhibited the highest growth rate at 37 °C (Fig. 1). *B*. *pseudomallei* grew at 30 °C, 40 °C and 42 °C, albeit at a lower rate. The average doubling times at log phase were 97.7, 90.1, 46.1, 59.5 and 84.0 min for 25 °C, 30 °C, 37 °C, 40 °C and 42 °C, respectively (25 °C versus 37 °C, P = 0.004; 30 °C versus 37 °C, P < 0.001; 37 °C versus 40 °C, P = 0.001; 37 °C versus 42 °C, P = 0.001). The number of viable bacteria cultured at 42 °C gradually fell over time after 24 h of incubation. Exponential growth was delayed until 12 h of incubation for *B*. *pseudomallei* cultured at 25 °C, with stationary phase ultimately reached at 48 h. These data indicate that *B*. *pseudomallei* can grow and survive in a broad range of temperatures.

### Proteomic alteration in *B*. *pseudomallei* under heat stress conditions

**Figure 2:**
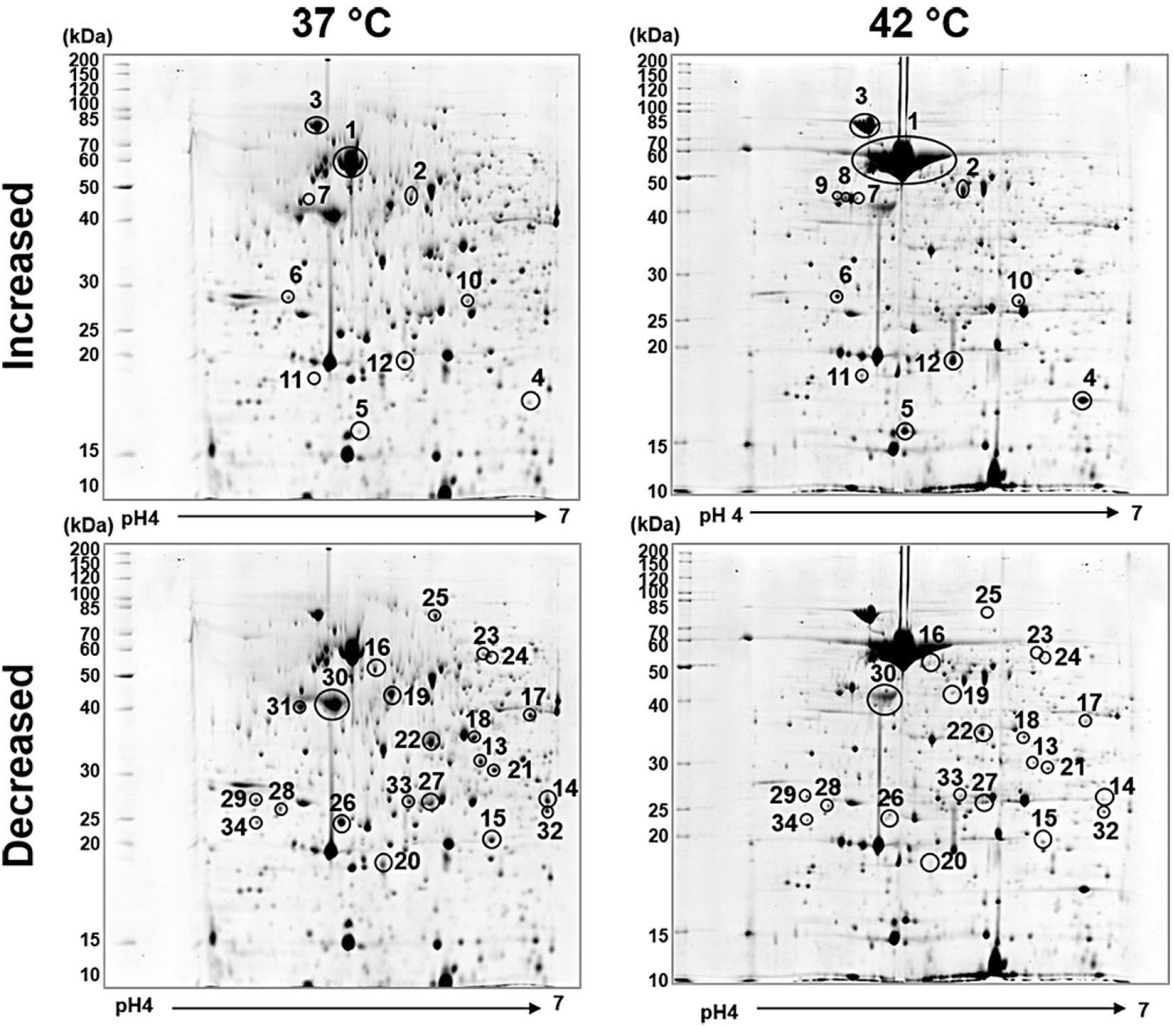
Proteomic profiles of *B*. *pseudomallei* strain 153. Circles are used to highlight up-regulated proteins (top) and down-regulated proteins (bottom) in bacteria cultured at 42 °C (right) compared with those cultured at 37 °C (left). Protein spot numbers related to information provided in Table 1.

We analysed the proteomic changes of *B*. *pseudomallei* strain 153 grown at 37 °C and 42 °C by two-dimensional (2D) gel electrophoresis. Approximately 600 protein spots were visualised after Coomassie blue staining, quantitative intensity analysis of which revealed 34 differentially expressed protein spots with ≥ two-fold change between *B*. *pseudomallei* cultured at 42 °C versus 37 °C (Fig. 2 and Table 1). Of these, 12 had increased intensity (2.2 to 24.8 fold) and 22 had decreased intensity (2.3 to 19.0 fold). Two spots were present only at 42 °C, and one spot was absent at this temperature. These proteins were identified using matrix-assisted laser desorption/ionization time-of-flight mass spectrometry (MALDI-TOF MS) and were then categorised based on main biological function using UniProt Knowledgebase (Swiss-Prot and TrEMBL entries) (Table 1). Proteins with altered expression were identified as chaperones (7 spots), metabolic enzymes (12 spots), antioxidants (10 spots), motility proteins (2 spots), structural proteins (2 spots) and a hypothetical protein (1 spot).

**Table 1:** Summary of altered protein expression of *B*. *pseudomallei* strain 153 cultured in LB broth at 42 °C compared to 37 °C.

Protein name	Protein ID	Locus ID	Gene	Biological function	Spot number	MASCOT score	Mass (Da)	PI	Fold change
**Upregulated proteins at 42 °C**									
**Chaperones**									
60 Kda chaperonin	Q9F712	BPSL2697	*groEL*	protein folding/stress response	1	134	57137	5.13	2.97
					2	159	57137	5.13	2.73
Chaperone DnaK	O68191	BPSL2827	*dnaK*	protein folding/stress response	3	200	69773	4.94	2.72
Peptidyl-prolyl cis-trans isomerase B	Q63SS5	BPSL2246	*ppiB*	protein folding	4	210	17890	6.39	17.82
Heat shock HSP-20 related protein	Q63QV6	BPSL2918	—	stress response	5	89	16053	5.18	24.83
Chaperone protein HchA	A0A0H2WBH2	BMAA1406	*hchA*	stress response	6	213	23932	4.72	2.77
**Metabolic enzymes**									
Serine-type carboxypeptidase protein	A3N570	BMA2026	*cbp2*	proteolysis and cellular protein catabolic process	7	56	66090	5.77	3.67
Serine-pyruvate aminotransferase	Q63NF6	BPSS0343	*phnW*	phosphonate and phosphinate metabolism	8	101	38917	6.30	Present
Phosphoserine aminotransferase	Q63S02	BPSL2519	*serC*	glycine, serine and threonine metabolism	9	134	39425	5.94	Present
**Antioxidants**									
Ferredoxin-NADP(H) reductase	Q63YE7	BPSL0241	*fpr*	antioxidant enzyme	10	155	28983	5.78	2.22
Hydroperoxidase reductase	Q63T73	BPSL2096	*ahpD*	antioxidant enzyme	11	87	20463	5.05	2.76
AhpC/Tsa family, antioxidant	Q62I24	BMA2066	*tsa1*	antioxidant	12	88	23904	5.75	3.63
**Down regulated protein at 42 °C**									
**Chaperone**									
Universal stress protein family domain protein	Q2T4Y8	BTH_II1566	*uspA*	stress response	13	129	30371	5.84	−5.22
**Metabolism enzymes**									
Acetoacetyl-CoA reductase	Q63J00	BPSS1916	*phbB*	poly-hydroxybutyrate biosynthetic process	14	190	26583	6.30	−4.10
D-beta-hydroxyburyrate dehydrogenase	Q62CL0	BMAA0017	*bdhA-1*	ketone body biosynthetic process	15	83	28137	5.90	−5.78
ATP synthase F1, beta subunit	Q63IW3	BMA2957	*atpD-2*	ATP hydrolysis coupled proton transport	16	131	50819	5.26	−6.41
Elongation factor Tu	Q63PZ6	BPSL3215	*tuf*	protein biosynthesis	17	150	43192	5.36	−3.47
Non-ribosomally encoded peptide/ polyketide synthase	Q63L25	BPSS1183	*phyH*	biosynthetic process	18	102	35611	5.77	−2.63
Phosphoglycerate kinase	Q63WU5	BPSL0796	*pgk*	carbohydrate metabolic process	19	186	41379	5.58	−6.32
Inorganic pyrophosphatase	Q63W67	BPSL1021	*ppa*	phosphate-containing compound metabolic process	20	88	19206	5.37	−19.03
Rhodanese-related sulfotransferase	Q63JF0	BPSS1766	*sseA*	thiosulfate sulfurtransferase activity	21	127	31119	5.98	−3.82
Carbamate kinase	Q63U71	BPSL1745	*arcC*	arginine metabolic process	22	129	33507	5.54	−2.27
**Antioxidants**									
Catalase HPII	Q63I56	BPSS2214	*katE*	hydrogen peroxide catabolic process/stress response	23	152	78455	5.89	−6.80
					24	130	78455	5.89	−4.19
Catalase-peroxidase proteins	Q939D2	BPSL2865	*katG*	hydrogen peroxide catabolic process/stress response	25	215	81824	5.89	−13.67
Oxidoreductase	Q63RC4	BPSL2748	*tftC*	antioxidant enzyme	26	172	23904	5.75	−4.44
					27	95	23904	5.75	−3.22
Thioredoxin	Q63WN5	BPSL0856	—	antioxidant enzyme/redox homeostasis	28	233	33259	4.55	−3.64
Thioredoxin protein, putative	Q63WN5	BPSL0856	*trxA*	antioxidant enzyme/redox homeostasis	29	62	33352	4.71	−4.12
**Motility**									
Flagellin	H7C7G3	BPSL3319	*fliC*	bacterial flagella	30	106	39233	5.05	−2.60
					31	120	39233	5.05	Absent
**Structure**									
OmpA family protein	Q62M19	BMA0436	*ompA*	structural molecule/ion transmembrane transport	32	109	24291	9.51	−2.61
Chitin-binding protein, putative	Q63PN3	BPSL3340	*cbp*	structural molecule	33	110	25944	6.23	−2.94
**Miscellaneous**									
Hypothetical protein	Q63Y40	BPSL0348	—	—	34	60	28041	6.59	−2.39

**Figure 3:**
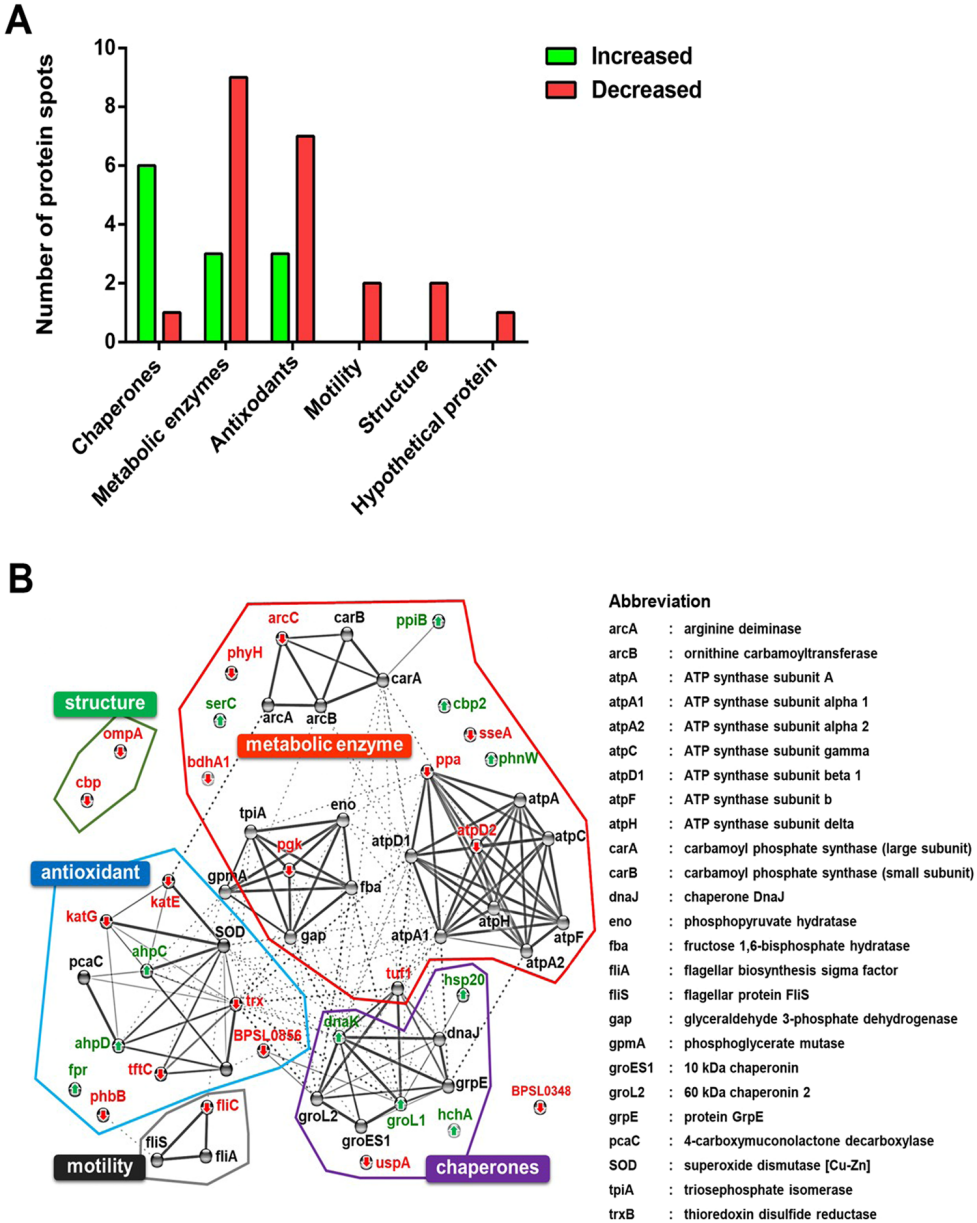
Altered proteins of *B*. *pseudomallei* cultured in LB broth at 42 °C under static conditions for 18 h. The proteins were categorised based on main biological functions by UniProt Knowledgebase (Swiss-Prot and TrEMBL entries) (**A**) and global protein network by STRING (**B**).

The 22 proteins with decreased expression at 42 °C were metabolic enzymes (9 spots), antioxidants (7 spots), bacterial motility (2 spots), structural proteins (2 spots), a chaperone (1 spot) and a hypothetical protein (1 spot) (Fig. 3A). By contrast, the 12 proteins with increased expression at 42 °C were mostly related to heat shock protein families and chaperones (6 spots), antioxidants (3 spots) and metabolic enzymes (3 spots) (Fig. 3A). Analysis of global protein networks using STRING revealed associations between these proteins produced at different levels (Fig. 3B), which were consistent with the functional categories derived from the UniProt Knowledgebase database (Table 1).

### RNA expression of *B*. *pseudomallei* under heat stress conditions

**Figure 4:**
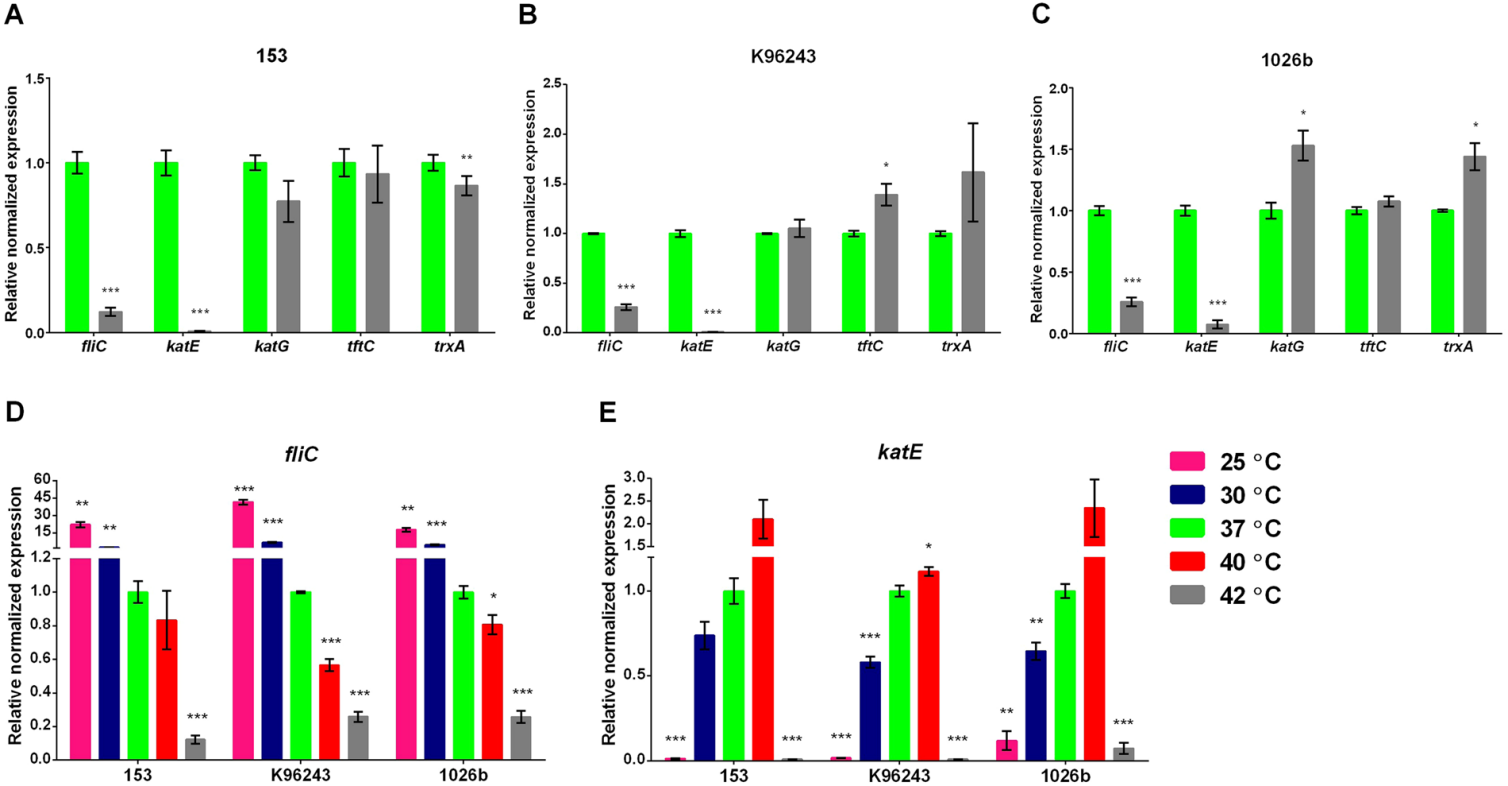
qRT-PCR relative expression levels of *fliC*, *katE*, *katG*, *tftC* and *trxA* in *B*. *pseudomallei* strains 153 (**A**), K96243 (**B**) and 1026b (**C**). (A), (B) and (C) *B*. *pseudomallei* was cultured in LB broth at 37 °C and 42 °C for 18 h. (**D**) and (**E**) *B*. *pseudomallei* was cultured in LB broth at 25 °C, 30 °C, 37 °C, 40 °C and 42 °C for 18 h. *16S* rDNA was used as a reference for the calculation of relative expression levels of other genes. The normalised expression levels were calculated by using 2^−ΔΔCt^ method^43^. Data represent the mean, and error bars represent the standard deviation. *P < 0.05; **P < 0.01, ***P < 0.001, for 37 °C versus other temperatures.

Of the multiple proteins altered by growth at 42 °C, redundancy in motility and antioxidant protein spots were observed among down-regulated proteins. To confirm these proteomic results, qRT-PCR was performed with three clinical isolates of *B*. *pseudomallei* from patients with melioidosis, strains 153, K96243 and 1026b for a motility gene (*fliC)*, antioxidant protein-coding genes (*katE*, *katG*, *tftC* and *trxA*) and a reference gene (*16S* rDNA*)* (Fig. 4A–C). All three bacterial strains demonstrated significant down-regulation of *fliC* and *katE* expression at 42 °C compared with 37 °C, while reduction in *katG*, *tftC* and *trxA* expression was variable between the three strains. To further validate the effect of temperature, we analysed the expression of *fliC*, *katE* and *16S* rDNA genes for *B*. *pseudomallei* cultured in LB broth at 25 °C, 30 °C, 37 °C, 40 °C and 42 °C for 18 h. All three strains showed highest *fliC* expression at 25 °C followed by 30 °C, 37 °C, 40 °C and 42 °C (Fig. 4D). In contrast, these three isolates had highest *katE* expression at 37 °C and 40 °C and reduced *katE* expression at other temperatures (Fig. 4E).

### Motility of *B*. *pseudomallei* at different temperatures

**Figure 5:**
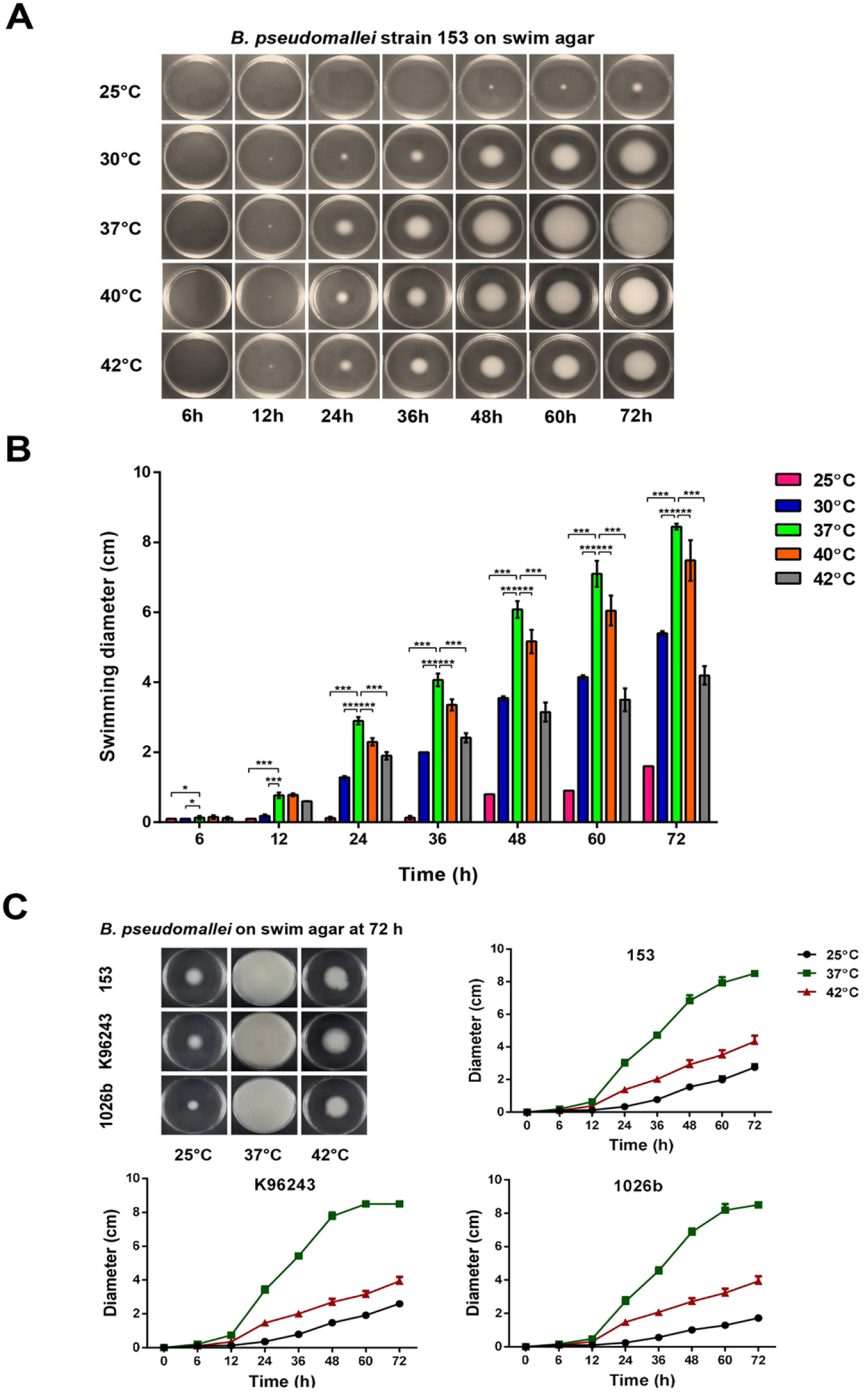
Swimming zone diameters of *B*. *pseudomallei* after incubation at different temperatures. (**A**) Swimming zones of *B*. *pseudomallei* strain 153 after incubation between 25 °C and 42 °C at different time points between 6 and 72 h, (**B**) Swimming zone diameters of *B*. *pseudomallei* strain 153 after incubation, (**C**) Swimming zone diameters of *B*. *pseudomallei* strains 153, K96243 and 1026b after incubation at 25 °C, 37 °C and 42 °C at different time points. The data represent the mean, and error bars represent the standard deviation.

We hypothesised that *B*. *pseudomallei* reduces motility and anti-oxidative stress functions to maintain synthesis of vital proteins for growth at high temperatures. The thermal effect on swimming motility was investigated at a range of temperatures for strain 153. Swim plates were incubated at 25 °C, 30 °C, 37 °C, 40 °C and 42 °C for 72 h. The zones of *B*. *pseudomallei* increased after incubation at all temperatures; the largest zone diameter was observed at 37 °C, followed by 40 °C, 42 °C and 30 °C, whereas the smallest zone diameter was observed at 25 °C (Fig. 5A,B). We re-examined swimming motility of bacteria at 25 °C, 37 °C and 42 °C for strains 153, K96243 and 1026b and obtained the same results (Fig. 5C). The swimming zone diameters of these strains increased overtime for all temperatures, with the largest diameters observed at 37 °C, followed by 42° and 25 °C. The diameters of all three strains after incubation at 25 °C and 42° were significantly lower than at 37 °C for all time points between 24 and 72 h (P < 0.001 for all strains, at 25 °C versus 37 °C and at 42 °C versus 37 °C).

**Figure 6:**
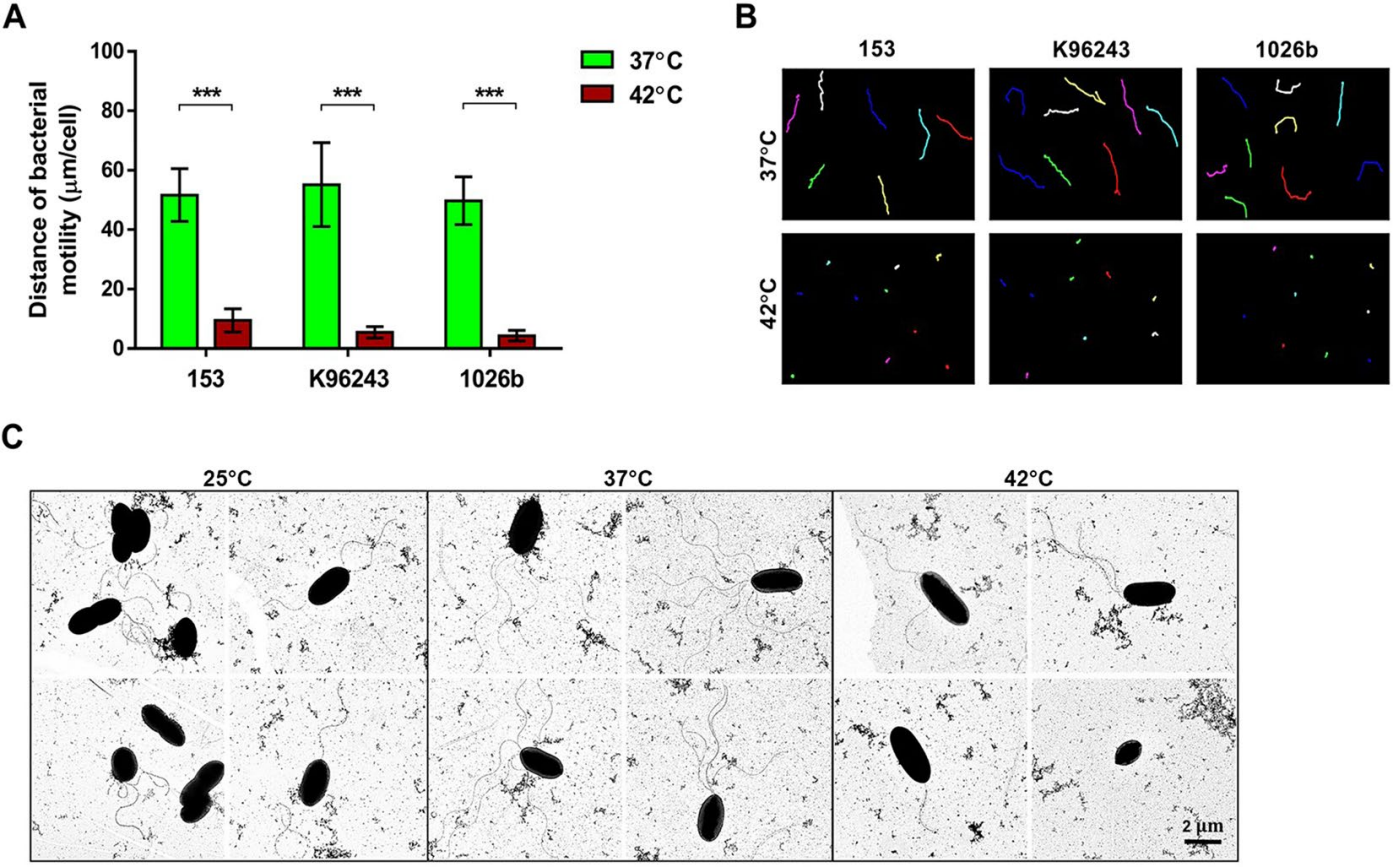
Live cell imaging analysis of individual *B*. *pseudomallei* cells for strains 153, K96243 and 1026b cultured at 37 °C and 42 °C for 6 h (**A**), and distance of bacteria cell motility (**B**). Motility was tracked for 20 s in 20 individual cells using ImageJ program (http://rsb.info.nih.gov/ij/). (**C**) Transmission electron microscopy of *B*. *pseudomallei* strain 153 cultured in LB broth at 25 °C, 37 °C and 42 °C for 6 h. ***P < 0.001.

To determine whether the reduced swimming zone at 42 °C was a result of reduced motility or growth, video-assisted imaging under light microscopy was employed to examine motility of individual bacterial cells grown in LB broth for 18 h. The computational measurement data demonstrated that the average distance of bacterial motility of the three *B*. *pseudomallei* strains was significantly lower when incubated at 42 °C compared with 37 °C (Fig. 6A,B). In addition, *B*. *pseudomallei* cultured at 25 °C and 37 °C demonstrated forward directional movement, but when cultured at 42 °C showed rotating or circular movements. These data indicate that temperature affects the motility of *B*. *pseudomallei*.

### Electron microscopy of *B*. *pseudomallei* under heat stress conditions

To understand the reduced swim motility of *B*. *pseudomallei* when cultured in LB broth for 6 h at 25 °C and 42 °C, we investigated the presence and number of flagella expressed by bacteria at these temperatures. Transmission electron microscopy confirmed a reduction in the proportion of flagellated bacteria at 42 °C (35%) compared with 37 °C (100%) and 25 °C (100%). The flagella of *B*. *pseudomallei* cultured at 42 °C appeared to be truncated. The mean (±standard deviation) number of flagella at 37 °C (3.3 ± 1.7, 95% CI 2.8–3.8) was significantly higher than those at 42 °C (1.6 ± 1.4, 95% CI 1.2–2.0) (P < 0.001) and 25 °C (2.1 ± 0.91, 95% CI 1.8–2.4) (P < 0.001) (Fig. 6C). These results showed that temperature changes affect the structure and expression of flagella in *B*. *pseudomallei*.

### Susceptibility of *B*. *pseudomallei* to reactive oxygen intermediates at different growth temperatures

**Figure 7:**
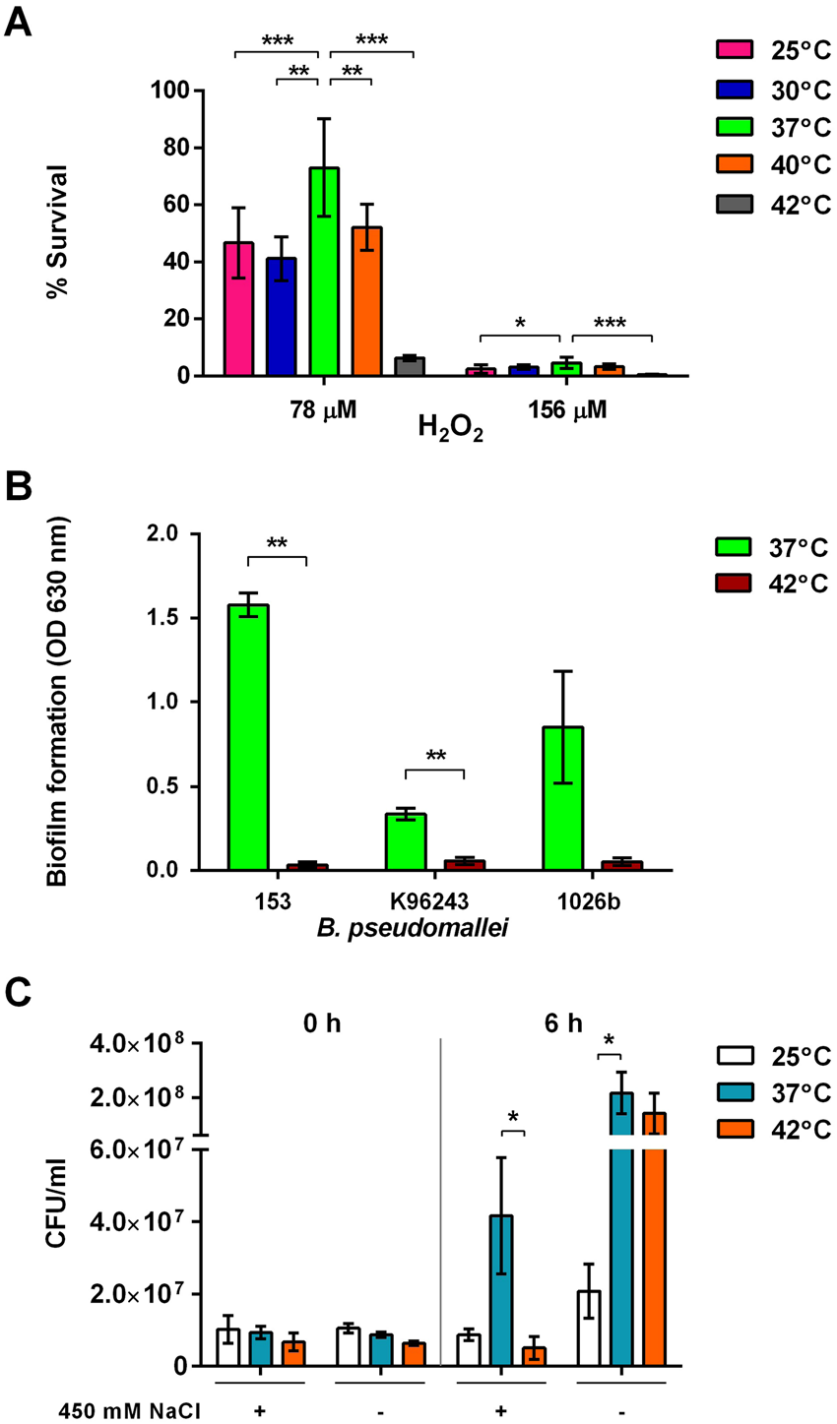
(**A**) Susceptibility of *B*. *pseudomallei* strain 153 to 78 µM and 156 µM H_2_O_2_ at different temperatures for 16 h. The number of colonies on oxidant agar plates containing H_2_O_2_ was normalised with those from plates without H_2_O_2_ and presented as the percentage of bacterial survival. (**B**) Biofilm formation of *B*. *pseudomallei* strains 153, K96243 and 1026b in LB broth for 48 h at 37 °C and 42 °C. The biofilm on a 96-well plate stained with 1% crystal violet was measured the OD at 630 nm. (**C**) Survival of *B*. *pseudomallei* under salt stress condition. *B*. *pseudomallei* were incubated at 25 °C, 37 °C and 42 °C for 18 h. The colony count was determined after exposure to 450 mM NaCl at 0 and 6 h. *B*. *pseudomallei* incubated at 25 °C, 37 °C and 42 °C in LB broth without added salt were used as the control. *P < 0.05; **P < 0.01, ***P < 0.001.

Proteome analysis and qRT-PCR of *B*. *pseudomallei* cultured at 42 °C demonstrated a pronounced decrease in expression of many antioxidant proteins. Because *katE* and *katG* encodes for catalases and peroxidase^21–23^, which are involved in H_2_O_2_ degradation, we tested the susceptibility of *B*. *pseudomallei* strain 153 to reactive oxygen intermediates (ROI) when cultured at different temperatures. The susceptibility of bacteria to ROI was investigated on LB agar plates containing 78 or 156 μM H_2_O_2_. Susceptibility varied depending on the growth temperature. *B*. *pseudomallei* cultured at 37 °C had the highest resistance to H_2_O_2_. In the presence of 78 μM H_2_O_2_, the most resistant bacteria were obtained from a culture at 37 °C followed by 40 °C, 25 °C and 30 °C. *B*. *pseudomallei* was most sensitive to H_2_O_2_ at 42 °C. The cell viability was low for bacteria cultured on the oxidant plate with 156 μM H_2_O_2_ (Fig. 7A)_._ The same results were obtained for K96243 and 1026b. These data suggest that temperatures can regulate the expression of genes encoding antioxidant enzymes in *B*. *pseudomallei*, and that these enzymes may be involved in the resistance to ROI.

### Biofilm-forming activity of *B*. *pseudomallei*

Biofilm formation has been previously reported to be associated with relapse in patients with melioidosis^24^. We, therefore, examined the effect of temperature on the biofilm-forming activity of *B*. *pseudomallei* strains 153, K96243 and 1026b in LB broth at 37 °C and 42 °C. Biofilm activity was significantly higher for all strains when cultured at 37 °C compared with 42 °C (Fig. 7B). The mean biofilm-forming activity varied between individual isolates. When cultured in LB broth at 42 °C, all strains failed to produce biofilm.

### Susceptibility of *B*. *pseudomallei* to salt

We previously demonstrated that *B*. *pseudomallei* can increase thermal tolerance after exposure to a high concentration of NaCl^13^. In the present study, we further examined the susceptibility of bacteria to a high salt concentration following heat treatment for 6 h. Susceptibility of the three *B*. *pseudomallei* strains to salt stress was tested with pre-incubation at 25 °C, 37 °C and 42 °C for 18 h prior to exposure to 450 mM NaCl in LB broth at the same temperatures for 6 h. The average numbers of viable bacteria at all temperatures were lower for bacteria cultured in LB broth with high salt compared with the bacteria cultured in LB without added salt as the control (Fig. 7C). In LB broth with high NaCl concentration, the average number of viable bacteria was highest for bacteria cultured at 37 °C with a bacterial count of 4.17 × 10^7^ CFU/ml, which was significantly different from bacteria cultured at 42 °C with a bacterial count of 5.06 × 10^6^ CFU/ml (P = 0.047). In LB broth without added salt as the control, the average number of viable bacteria cultured at 37 °C was not significantly different from that of 42 °C (2.17 × 10^8^ CFU/ml versus 1.42 × 10^8^ CFU/ml, P = 0.102) but was significantly higher than that of 25 °C (2.08 × 10^7^ CFU/ml, P = 0.049). These data confirm the effect of temperature on the resistance of *B*. *pseudomallei* to salt stress.

## Discussion

Melioidosis is a public health burden in tropical countries and an emerging disease that is estimated to cause 89,000 deaths globally each year^2^. Patients with melioidosis present with abscess and protracted fever that requires prolonged treatment. Our study focused on the effect of temperatures that are encountered by *B*. *pseudomallei* in the environment and the human host during infection. Our data demonstrated that *B*. *pseudomallei* could grow and survive in a wide range of temperatures. Thermal changes were found to trigger several responses in *B*. *pseudomallei* at the proteomic level. The proteins produced by *B*. *pseudomallei* due to thermic change included metabolic enzymes, chaperones, cell motility proteins, antioxidants, and structural proteins. These proteins were particularly produced by *B*. *pseudomallei* at 37 °C, a temperature which was found to support bacterial growth, motility, biofilm formation and resistance to oxidative stress.

The rapid growth of *B*. *pseudomallei* at 37 °C suggests that *B*. *pseudomallei* may proficiently replicate upon entry into hosts. The ability of the bacteria to grow at 38 °C–40 °C suggests that *B*. *pseudomallei* are capable of replicating in a host with a high fever. The range of temperatures used here of 25 °C–42 °C are similar to environmental conditions in tropical regions that support *B*. *pseudomallei* in soil and water. Indeed, it has been found that *B*. *pseudomallei* can remain viable with high bacterial numbers of more than 10,000 CFU per gram of soil in northeast Thailand^3,4,25^. Although the growth of *B*. *pseudomallei* is reduced at lower temperatures such as 25 °C, stationary phase is finally achieved and cell viability remains high. However, our study demonstrated that *B*. *pseudomallei* grew at a high rate at 42 °C but viability slowly declined after stationary phase if the high temperature was maintained. Our results suggested that the dynamics of temperature changes during the day and night or in different seasons may affect the growth of *B*. *pseudomallei*, but that the bacterium can tolerate fluctuation in environmental temperature in tropical climates.

Different growth temperatures had a broad impact on protein expression by *B*. *pseudomallei*. Proteomic analysis identified up- and down-regulated proteins in response to temperature changes, suggesting that temperature is an important environmental signal for cellular metabolism aimed at maintaining the growth and biological activity of the bacterium. Many studies have shown that alteration of bacterial cellular proteins is a necessary mechanism for cell survival under heat stress^15,16,26^. During early exposure to high temperatures, reactive oxygen species (ROS) are generated, and there is an accumulation of misfolded membrane proteins. Our result demonstrated that at 42 °C, *B*. *pseudomallei* had reduced levels of outer membrane proteins (OmpA) and chitin-binding proteins (CBPs). OmpA is a structural and immunogenic protein^27,28^ and CBPs of many bacterial pathogens contain surface-exposed domains that support infection of non-chitinous mammalian hosts^29^. The increased levels of many chaperones at high temperatures are consistently observed in other bacteria^17,19^. Heat shock proteins (HSPs) are well known to participate in protein folding, refolding and removal of non-functional damaged proteins^16,18^. *Escherichia coli* respond to high temperatures by activating a heat shock response. The stability of mRNAs of heat shock genes has been shown to be maintained by the cold shock protein C (CspC)^15^, but this mechanism is still unknown for *B*. *pseudomallei*.

An adaptive mechanism utilised by bacteria to survive at elevated temperatures is the reduction of metabolic activity, which is crucial for energy conservation^30^. Our findings confirmed that expression of several metabolic proteins including acetoacetyl-CoA reductase, D-beta-hydroxybutyrate dehydrogenase, ATP synthase, elongation factor Tu, non-ribosomally encoded peptide/polyketide synthase, phosphoglycerate kinase, inorganic pyrophosphatase, rhodanese-related sulfotransferase and carbamate kinase were decreased. We previously showed that carbamate kinase encoded by *arcC* is a component of the arginine deiminase system, which facilitates acid tolerance in *B*. *pseudomallei*^12^. The decreased level of carbamate kinase under heat stress would potentially lead to defective cell survival processes. However, some cellular metabolic proteins were upregulated, including carboxypeptidase protein, pyruvate aminotransferase and phosphoserine aminotransferase.

In our study, qRT-PCR analysis confirmed the proteomic results in the modification of transcription levels of *fliC* (encoding flagellin) and *katE* expression by temperature changes. Experiments on three *B*. *pseudomallei* strains using swim agar validated the proteomic results that motility and the presence of flagella were affected by temperature change, with the highest function observed at 37 °C. Temperature-dependent regulation of flagella expression was previously reported in *B*. *thailandensis*, wherein bacterial growth at 28 °C enhanced cell motility and flagella expression through a mechanism involving the regulation of the *fliC* gene at the mRNA stability level^31^. *B*. *thailandensis* is a non-virulent environmental species of *Burkholderia* which is closely related to *B*. *pseudomallei*. We observed similar transcriptomic results in *B*. *pseudomallei* that *flicC* expression was highly expressed at 25 °C. However, our results were based on three *B*. *pseudomallei* strains that showed larger swimming zones at 37 °C compared with those at 25 °C and 30 °C. The discrepancy between transcriptomic results and swimming zone at 25 °C and 30 °C may be associated with bacterial growth rate or a complex process of a translation inhibition of FliC, an issue that requires further investigation. Our data from live cell imaging and electron microscopy also confirmed that the large zone diameter at 37 °C was associated with individual cell movement and flagella production. However, swimming motility was reduced when *B*. *pseudomallei* were cultured at 42 °C, which was consistent with proteomic and mRNA transcription of the *fliC* gene. The proteomic analysis suggested that response to increased temperature may enable bacterial cells to produce more important stress response proteins, namely chaperones and metabolic enzymes and antioxidants. Temperature has been reported to regulate flagellar motility genes in other bacteria such as *Pseudomonas syringae*^32^ and *Listeria monocytogenes*^33^.

We also found that *B*. *pseudomallei* underwent a number of phenotypic adaptations under different growth temperatures. Altered flagella expression could be linked to the virulence and modulation of the immune response during infection. French *et al*. demonstrated that bacterial flagella support rapid intracellular motility and efficient cell-to-cell spread, thus forming multinucleate giant cells (MNGC), leading to cell death^34^. A notable virulence feature of *B*. *pseudomallei* is the formation of MNGC, which occurs in many cell types including phagocytic and non-phagocytic cells^35,36^. Flagellin (FliC) of *B*. *pseudomallei* is an important innate immune ligand for Toll-like receptor (TLR) 5. It activates NF-κB and leads to the production of inflammatory cytokines^37^. Our data highlight the importance of flagellin expression, which may be transiently regulated by the alteration of host temperature.

All three *B*. *pseudomallei* strains were efficiently resistant to ROI, with the highest resistance observed when cultured at 37 °C. This observation on oxidant agar was consistent with the results of proteomic and transcriptional analyses showing high expression of *katE* at 37 °C and 42 °C compared with those at 42 °C and other temperatures. *katE* (BPSS2214) and *katG* (BPSL2865) encode for catalase and peroxidase enzymes, respectively, which catalyse H_2_O_2_ degradation. These enzymes are important for the survival of facultative aerobic organisms exposed to oxidative stress. *katG* and *katE* expression in *B*. *pseudomallei* has also been reported to be regulated by *rpoS* and *oxyR*^38^, whereas *katE* expression is regulated by *rpoN2*^39^ in response to several stress conditions, such as nutritional starvation and oxidative stress. Further studies are required to investigate the effect of temperature on the expression and roles of these regulators.

Our data demonstrated that biofilm activity was significantly higher for all three strains when cultured at 37 °C compared with 42 °C. Consistent with our study, the effect of temperature on biofilm formation has recently been reported in *B*. *pseudomallei*, in which the level of biofilm formation by *B*. *pseudomallei* strain 1026b in LB medium decreased ∼6-fold at 40 °C and 42 °C compared with 37 °C^40^. The role of temperature in the regulation of biofilm formation has been linked to cyclic di-GMP signaling in *B*. *pseudomallei*^40^. Our data suggest that biofilm formation is associated with *B*. *pseudomallei* cells that are more metabolically active during growth at 37 °C. The decrease in biofilm seen at 42 °C was possibly related to the down-regulation of the flagella expression and other metabolic enzymes.

We previously showed that a high NaCl concentration affects the survival and adaptive mechanisms of *B*. *pseudomallei*. In response to exposure to high NaCl concentration, *B*. *pseudomallei* isolates show an increased thermal tolerance, oxidative resistance, and plaque-forming efficiency^13^. However, the result of the present study demonstrated that *B*. *pseudomallei* had the greatest tolerance to salt stress following pre-incubation at 37 °C in comparison with that at 25 °C and 42 °C. Increased temperature of 42 °C was negatively associated with reduced bacterial viability under the same salt stress conditions. Resistance to salt at 37 °C may provide a major benefit for *B*. *pseudomallei* stability in environmental niches where the bacteria encounter a warm temperature but high salt concentrations.

Our study suggested that the human body temperature of 37 °C facilitates maximum growth rate, motility and anti-oxidative stress functions in *B*. *pseudomallei*. These mechanisms may be a prerequisite for acute infection and dissemination. In conditions with high temperatures, *B*. *pseudomallei* expresses a number of stress-response proteins but decreases its motility by reducing flagella expression. This may ultimately lead to latent infection if the patients do not recover from melioidosis.

## Materials and Methods

### Ethics statement

The study was approved by the Ethics Committee of the Faculty of Tropical Medicine, Mahidol University (approval number: MUTM2018-009-01). All bacterial isolates obtained from humans were anonymous.

### *B. pseudomallei* strains

Three *B*. *pseudomallei* isolates from patients with melioidosis in northeast Thailand were used: strains 153, 1026b and K96243. All isolates grew with a type I colony morphology on Ashdown agar after incubation for 4 days at 37 °C in air^41^. Unless otherwise stated, a single colony of *B*. *pseudomallei* was cultured on LB, Lennox agar or broth and incubated at 37 °C in air for 18 h before being used in each experiment.

### Bacterial growth curve analysis

Bacterial culture was performed in a BSL-3 laboratory. Several colonies of *B*. *pseudomallei* were suspended in sterile PBS and the optical density (OD) at 600 nm adjusted to obtain a bacterial concentration of approximately 1 × 10^8^ colony-forming units (CFU)/ml. Five microliters of bacterial suspension was added to 5 ml of LB broth to make a final concentration of 1 × 10^5^ CFU/ml. Cultures were then incubated in duplicate at five different temperatures under static conditions: 25 °C, 30 °C, 37 °C, 40 °C and 42 °C and sampled at time intervals (0-, 6-, 12-, 24-, 36-, 48-, 60- and 72 h), aliquots from which were serially diluted in sterile PBS. Ten microliters of each dilution were inoculated on Columbia agar in triplicate, followed by incubation at 37 °C in air for 16 h before a colony count was performed.

### Protein extraction

A single colony of *B*. *pseudomallei* on Ashdown agar was picked and inoculated into 100 ml of LB broth, which was incubated at 37 °C or 42 °C under static conditions for 18 h. Two independent experiments were performed. Bacteria were harvested by centrifugation at 4,500 × g for 30 min followed by washing with PBS. The bacterial pellet was resuspended in 1 ml of a cold lysis buffer (5 mM EDTA and 1 mM phenylmethylsulfonyl fluoride)^12^ and then sonicated on ice at 22% amplitude for 3 min. The cell lysate was centrifuged at 14,000 × g at 4 °C for 3 min, and the supernatant was then collected and filtered using a 0.2-μm filter. Protein samples were stored at −80 °C until use.

### 2D gel electrophoresis and quantitative spot intensity analysis

After cleaning the protein samples, protein concentration was measured using a 2D quantification kit (GE Healthcare Bio-Sciences). Protein complexes (700 µg) derived from two independent experiments for each condition were separated by isoelectric focusing on Immobiline DryStrip (linear pH gradient of 4–7, 18 cm) followed by 12% polyacrylamide gel electrophoresis, as previously described^12^. Separated protein spots were visualised with colloidal Coomassie blue G-250 stain and then captured using an Image Scanner II and LabScan software version 5.0 (GE Healthcare Bio-Sciences). Image Master 2D Platinum software 7.0 (GE Healthcare Bio-Sciences) was used for matching and analysis of the protein spots in 2D gels. The fold change in the intensity of matched protein spots between 37 °C and 42 °C was calculated. The differential spots representing more than a two-fold change were then subjected to protein identification by mass spectrometry.

### In-gel tryptic digestion and protein identification by MALDI-TOF MS

Candidate proteins were excised from the gel using the Ettan Spot Handling Workstation (GE Healthcare Bio-Sciences) followed by digestion with trypsin, as previously described^12^. The trypsinized samples were deposited onto the 96-well MALDI target plate (MTP 384 polished steel TF), and protein mass spectra were obtained using an Autoflex MALDI-TOF MS (Bruker Daltonik, Bremen, Germany).

### MASCOT searching and protein interaction network analysis

The MS data were exported as searchable files and used to search against peptide mass fingerprint in the MASCOT database (http://www.matrixscience.com). Protein identification was achieved using a protein BLAST search. All identified proteins were classified based on their main biological functions using UniProt Knowledgebase (Swiss-Prot and TrEMBL entries). The differentially expressed proteins isolated at 42 °C compared with at 37 °C were further analysed for protein interaction network using a STRING tool software (version 8.3) (http://string.embl.de/)^42^. Predicted protein–protein associations were queried through experimentally derived physical protein interactions from the research literature and databases of curated biological pathway knowledge.

### RNA extraction

*B*. *pseudomallei* strains K96243, 153 and 1026b were inoculated into 5 ml LB broth and incubated at 25 °C, 30 °C, 37 °C, 40 °C and 42 °C for 18 h under static conditions, after which bacteria were harvested by centrifugation at 12,000 rpm for 2 min. The pellet was washed with 1 ml of PBS, and RNA was extracted using RNeasy kit (Qiagen, Germany). The integrity of the purified RNA was examined by agarose gel electrophoresis, and the concentration was measured using a Nanodrop spectrophotometer (Thermo Scientific, USA). Contaminant genomic DNA was removed using DNase I treatment according to the manufacturer’s instructions (Thermo Scientific, USA), and the presence of residual DNA was checked by PCR using primers for the *16S* rDNA gene (Table S1).

### Quantitative reverse transcriptase PCR (qRT-PCR)

Two-step qRT-PCR was used to quantitatively measure gene expression using the iScript^TM^ Reverse Transcription Supermix for RT-qPCR (Bio-Rad, USA) and a CFX96 Touch ^TM^ Real-Time PCR Detection System with Bio-Rad CFX Manager software version 3.0 (Bio-Rad). All primer pairs were designed using NCBI PrimerBlast (https://www.ncbi.nlm.nih.gov/tools/primer-blast/). The primer sequences are shown in Table S1. The amplification was performed for the *fliC*, *katE*, *katG*, *tftC*, *trxA* and *16* *S* rDNA genes in duplicate in 10 µl total volume containing 5 µl of 2 iTaq Universal SYBR Green (Bio-Rad), 1 µl of cDNA, 0.4 µl of each primer (10 µmol/l), and 3.2 µl of distilled water. The cycle conditions were as follows: 1 cycle of 95 °C for 30 s followed by 40 cycles of 95 °C for 10 s and 60 °C for 30 s. After amplification, melting curve analysis was conducted by increasing the annealing temperature by 0.1 °C per step from 65 °C to 95 °C. The *16 S* gene was used as the reference for calculating the relative expression levels of other genes. The normalised expression levels were calculated by using the 2^−ΔΔCt^ method, where ΔΔCt = (Ct _target gene_ − Ct _*16S rDNA* gene_) at other temperatures − (Ct _target gene_ − Ct _*16S* rDNA gene_) at 37 °C^43^.

### Swimming motility assay

Two single colonies of *B*. *pseudomallei* were cultured in 5 ml of LB broth at a range of temperatures (25 °C, 30 °C, 37 °C, 40 °C and 42 °C) under static conditions for 18 h. The OD at 600 nm of the bacterial suspension was adjusted in sterile PBS to a concentration of 1 × 10^8^ CFU/ml and point inoculated into the centre of swimming agar plate (1% tryptone, 0.5% NaCl and 0.3% agar) using a sterile toothpick, as described previously^41^. The swim plates were further incubated at the same temperatures. The swimming zone diameters were measured in three different positions including the widest, medium and shortest diameters of the circular turbid zone formed by the bacterial cells migrating away from the point of inoculation at 0-, 6-, 12-, 24-, 36-, 48-, 60- and 72 h time intervals and the average zone diameters are presented.

### Live cell imaging of cell motility in *B*. *pseudomallei*

Live cell imaging was used to monitor the motility of *B*. *pseudomallei* using the hanging drop method. *B*. *pseudomallei* were cultured on Ashdown agar at 37 °C overnight. Colonies of each isolate were harvested and suspended in 10 ml of LB broth, and their OD at 600 nm was adjusted to a concentration of 1 × 10^10^ CFU/ml. Equal volumes of the bacterial suspension (2.5 ml) were added into two tubes containing LB broth to make a final volume of 5 ml and incubated separately at 37 °C and 42 °C for 6 h under static conditions to obtain log-phase cultures. Thereafter, 30 µl of the bacterial suspension was dropped on a glass slide with a depression, over which a cover slip was placed. Motility was observed using a light microscope (Leica DM750, Wetzlar, Germany) at 1000 × magnification. A video of the motility was recorded and the motility of 20 individual cells tracked for 20 s using ImageJ program (http://rsb.info.nih.gov/ij/) with the manual tracking plug-in.

### Transmission electron microscopy

*B*. *pseudomallei* strain 153 was cultured in LB broth at 25 °C, 37 °C and 42 °C for 6 h. A preparation of the bacterial cells was examined using transmission electron microscopy (TEM) for the presence of flagella, as previously described^44^. The bacterial cells were observed with a Hitachi Electron Microscope HT-7700 (Hitachi, Japan). The presence of bacterial flagella was recorded for 50 bacteria in each temperature.

### Determination of susceptibility of *B*. *pseudomallei* to reactive oxygen intermediates (ROI)

The susceptibility of *B*. *pseudomallei* to ROI at different temperatures was determined on oxidant agar plates, as previously described^7^. Briefly, *B*. *pseudomallei* were inoculated in 10 ml of LB broth and incubated at different temperatures (25 °C, 30 °C, 37 °C, 40 °C and 42 °C) for 16 h. The bacterial suspension from each condition was diluted with sterile PBS to make 10-fold dilutions. Ten microliters of a serially diluted inoculum were inoculated onto LB agar containing 0, 78 and 156 μM H_2_O_2_ in triplicate. The colonies were counted after incubation at 37 °C in air for 24 h. The number of colonies on the plates containing H_2_O_2_ was normalised with those from plates without H_2_O_2_ by presenting as the percentage of bacterial survival.

### Assessment of biofilm-forming activity of *B*. *pseudomallei*

The biofilm-forming activity of *B*. *pseudomallei* was assessed as described previously^41^. Briefly, the bacteria were inoculated into 10 ml of LB broth and incubated at 37 °C with shaking for 18 h. The OD at 600 nm of the bacterial suspension was adjusted to 1.0 with fresh LB broth and 200 µl of the suspension was added into a 96-well flat-bottomed plastic tissue culture plate (FALCON, USA), with eight wells/strain. Wells with only LB broth were included as negative controls. The plates were incubated at 37 °C or 42 °C to allow adhesion for 3 h. The supernatant was gently aspirated and replaced with 200 µl of fresh LB broth, and the plate was continually incubated at the same temperature for an additional 21 h. The supernatant was replaced with a fresh LB broth and incubated as above for an additional 24 h. Thereafter, the wells were washed with PBS, fixed with methanol for 15 min, and air-dried at room temperature. The plates were stained with 1% crystal violet and solubilised with 250 µl of 33% (v/v) glacial acetic acid per well before measuring the OD at 630 nm using a TECAN microplate reader (TECAN, Switzerland).

### Salt stress assay

The survival of *B. pseudomallei* strains K96243, 1026b and 153 under salt stress conditions were determined by observing the number of viable bacteria after exposure to 450 mM NaCl, as previously described^13^. Briefly, *B*. *pseudomallei* were incubated in 5 ml of LB broth at 25 °C, 37 °C and 42 °C for 18 h. The bacterial cells were harvested, washed and resuspended in PBS. The OD at 600 nm of the bacterial suspension was adjusted to 0.15 to obtain a bacterial concentration of 1 × 10^8^ CFU/ml. The bacteria were then inoculated at a dilution of 1:10 into 10 ml of LB broth containing 450 mM NaCl or LB broth without added salt as the control. The bacteria were further incubated at the same temperatures for 6 h with shaking. A 10-fold dilution of the bacteria was prepared and plated onto LB agar. After incubation at 37 °C for 24 h, colonies were counted. The mean numbers of colonies of the three *B. pseudomallei* strains were presented as the CFU.

### Statistical analysis

Statistical analyses were performed using Stata, version 12 (StataCorp LP, College Station, TX, USA). One-way ANOVA or Student *t*-test was used to test differences in quantitative data among different groups. All data in this study were presented as mean ± standard deviation. Differences were considered statistically significant at a P value < 0.05.

### Data availability

All data generated or analysed during this study are included in this published article.

## Electronic supplementary material

Table S1
