# Supplementary material for: Effect of temperature on Burkholderia pseudomallei growth, proteomic changes, motility and resistance to stress environments
Source: Sci Rep. 2018 Jun 15;8:9167. doi: 10.1038/s41598-018-27356-7 (PMC6004011; doi:10.1038/s41598-018-27356-7)
Supplement: Supplementary file 1 — Table S1 [file 41598_2018_27356_MOESM1_ESM.docx]

**Effect of temperature on *Burkholderia pseudomallei* growth, proteomic changes, motility and resistance to stress environments**

Suporn Paksanont^1^, Kitisak Sintiprungrat^1^, Thatcha Yimthin^1^, Pornpan Pumirat^1^, Sharon J. Peacock^1,2^, and Narisara Chantratita^1,3,*^

^1^Department of Microbiology and Immunology, Faculty of Tropical Medicine, Mahidol University, Bangkok, Thailand

^2^London School of Hygiene and Tropical Medicine, London, UK

^3^Mahidol-Oxford Tropical Medicine Research Unit, Faculty of Tropical Medicine, Mahidol University, Bangkok, Thailand

^*^Corresponding author: Narisara Chantratita. Department of Microbiology and Immunology, Faculty of Tropical Medicine, Mahidol University, 420/6 Rajvithi Road, Bangkok 10400, Thailand. E-mail: [narisara@tropmedres.ac](mailto:narisara@tropmedres.ac)

**Table S1.** Oligonucleotide primers used for real-time qRT-PCR

| Gene | Primer Sequencing (5’ 🡪 3’) |
| --- | --- |
| *fliC* | F_GCAGCAGATCTCGGAAGTGA  R_GACATGCTTTGCGTGAGGTC |
| *katE* | F_TGATTCCGGAGGAAACGGTG  R_GTCGGTGTACGAGAAGAGCC |
| *katG* | F_CAATCAGCTGGACCTGAGCA  R_GATGCCATGCGGATGAACAG |
| *tftC* | F_ATCGCAAGGTCTCGGAACTC  R_CGGGTAGGTGATGATGAGGC |
| *trxA* | F_ATCCAACATCGACGAGGACG  R_TCGACGTTCACCTTCACGAG |
| *16S* | F_GTGGGGAATTTTGGACAATG  R_CCGGGTATTAGCCAGAATGA |
